# Altered expression of antioxidant enzymes and autophagic proteins in transglutaminase 2 knockout mice

**DOI:** 10.1186/1750-1326-8-S1-P15

**Published:** 2013-09-13

**Authors:** Barbara D’Orio, Luana Barone, Anna Fracassi, Francesca Fanelli, Sara Sepe, Marco Segatto, Mauro Piacentini, Roberta Nardacci, Sandra Moreno

**Affiliations:** 1 Department of Science, University Roma Tre, Viale Marconi 446, 00146, Rome, Italy

## Background

Transglutaminases (TGs) are ubiquitous calcium-dependent acyl-transferases, catalysing post-translational protein modifications. Among the family members, TG2 (or “tissue” transglutaminase) acts as a multifunctional protein regulating cell processes, including autophagy [1].

TG2 is highly expressed in the nervous tissue and reportedly involved in neurodegenerative disorders. Indeed, the pathophysiology of these diseases includes insoluble aggregate formation, and covalent cross-linking of pathogenic proteins by TG2 has been suggested. Another hallmark of neurodegeneration is dysregulated autophagy, thus making the role played by TG2 in this cellular process especially relevant. Interestingly, conditions promoting TG2 activity, such as low GTP and high calcium levels, associated with oxidative stress, occur in neurodegeneration.

The present study aims to clarify the role of TG2 in redox balance and autophagy. To this purpose, the expression levels of antioxidant enzymes and pro-autophagic proteins were investigated in various brain regions and liver of TG2-/- mice.

## Materials and methods

Expression of antioxidant enzymes, namely superoxide dismutase 1 and 2 (SOD1, SOD2), catalase (CAT) and glutathione peroxidase 1 (GPX1), and autophagic proteins (Beclin1, LC3 and AMBRA1) were evaluated by Western blotting (WB) and immunohistochemistry in selected brain areas (neocortex, hippocampus, brainstem, and cerebellum) and liver of 12-month-old TG2-/- and *wild-type* mice.

## Results

WB and immunohistochemical data reveal altered expression patterns of antioxidant enzymes in both liver and brain tissues. Of particular interest is the statistically significant decrease of CAT and SOD2 in the cerebellum and hippocampus of TG2-/- mice, while unchanged levels of these proteins are detected in the neocortex and brainstem. In the knockout liver, even more dramatic reduction of CAT and SOD2 expression is found, while SOD1 is intriguingly upregulated. Concerning the effect of TG2 deletion on autophagy, Beclin1 is down-regulated in the neocortex and hippocampus of knockout mice.

## Conclusions

Overall, our data on TG2-/- mice support the involvement of the transamidating enzyme in controlling redox balance of different organs, and in regulating autophagic flux [1-3]. The specific decrease in the peroxisomal enzyme CAT and in the mitochondrial protein SOD2 emphasizes the role of these organelles in oxidative stress management and their interplay in cell metabolism. Moreover, region-based differences in the effect of TG2 deletion may reflect multiple functions related to the organ, tissue and cell type.
